# Natriuretic peptides as predictors for atrial fibrillation recurrence after catheter ablation: A meta-analysis

**DOI:** 10.1097/MD.0000000000033704

**Published:** 2023-05-12

**Authors:** Yujing Yuan, Boyuan Nie, Binbin Gao, Caixia Guo, Li Li

**Affiliations:** a Department of Cardiology, Shanxi Cardiovascular Hospital, Taiyuan, Shanxi, People’s Republic of China; b Department of Day Surgery, The Second Hospital of Shanxi Medical University, Taiyuan, Shanxi, People’s Republic of China.

**Keywords:** atrial fibrillation, catheter ablation, meta-analysis, natriuretic peptide, recurrence

## Abstract

Catheter ablation (CA) has become the first-line treatment strategy for atrial fibrillation (AF) but remains with a substantial recurrence rate. The aim of this meta-analysis was to determine the association between baseline natriuretic peptide levels and AF recurrence after CA.

**Methods:**

We systematically searched PubMed, EMBASE, Web of Science, and Wiley-Cochrane Library for relevant studies published up until May 2022. Overall effect analysis and subgroup analysis were performed with Review Manager software.

**Results:**

Finally, 61 studies that met the inclusion criteria were included in our meta-analysis. Compared with the nonrecurrence group, the recurrence group had increased baseline level of atrial natriuretic peptide (ANP) (standardized mean difference [SMD] = 0.39, 95% confidence interval [CI]: 0.21–0.56), brain natriuretic peptide (BNP) (SMD = 0.51, 95% CI: 0.31–0.71), N-terminal pro-BNP (SMD = 0.71, 95% CI: 0.49–0.92), and midregional N-terminal pro-ANP (SMD = 0.91, 95% CI: 0.27–1.56).

**Conclusions:**

Increased baseline natriuretic peptide levels, including ANP, BNP, N-terminal pro-BNP, and midregional N-terminal pro-ANP, are associated with a higher risk of AF recurrence after CA. Nonetheless, further studies are needed to elucidate the predictive value of baseline natriuretic peptides in AF patients undergoing CA.

## 1. Introduction

Atrial fibrillation (AF) is a common cardiac rhythm disturbance associated with serious complications including strokes, thromboembolism, and cognitive impairments that can influence patients’ quality of life and impose higher medical care costs.^[1]^ The disease remains an increasing public health concern throughout the world. Approximately 3.9 million individuals aged over 60 years suffer from AF in China.^[2]^ As a progressive disease, AF is associated with a higher risk of all-cause and cardiovascular death.^[3]^ Nowadays, catheter ablation (CA) is recommended as the first-line treatment strategy for rhythm control due to its effectiveness in sustaining sinus rhythm.^[4,5]^ However, it still has a substantial recurrence rate, and the success rate ranges from 30 to 85%.^[6]^ Therefore, how to judge the prognosis of CA is particularly important.

Natriuretic peptides are primarily secreted by myocytes under various stimuli and have been widely used as important diagnostic and monitoring tools for cardiovascular diseases.^[7]^ Meanwhile, studies have shown that AF patients have higher plasma natriuretic peptide concentrations than that in the control group, and the elevated natriuretic peptide levels decrease after CA restores sinus rhythm.^[8,9]^ However, whether increased natriuretic peptide is a true predictor of AF recurrence still remains controversial. Therefore, we conducted this meta-analysis to include more studies, discover different kinds of natriuretic peptides, and identify the association between baseline natriuretic peptide levels and AF recurrence after CA.

## 2. Methods

### 2.1. Search strategy

In order to aggregate all of the relevant published studies, Preferred Reporting Items for Systematic Reviews and Meta-Analyses guidelines were used for all peer-reviewed studies. We searched PubMed, EMBASE, Web of Science, and Wiley-Cochrane Library for relevant studies published up until May 2022. The following search items were used: natriuretic peptide, AF, CA, and recurrence. The “related article” function was also used during the search; the references for retrieved articles were manually searched to avoid initial misses.

### 2.2. Selection criteria

The included studies had to fulfill the following inclusion criteria: studies were designed as randomized controlled trials, prospective observational studies, or retrospective observational studies; the study enrolled patients who underwent their first CA; post-ablation AF recurrence was assessed as an outcome; atrial arrhythmia including AF, atrial flutter, or atrial tachycardia confirmed by 12-lead electrocardiography or Holter electrocardiography is used in the assessment of AF recurrence; and the number of patients, mean (standard deviation [SD]) or median (range or interquartile range) level of natriuretic peptides within recurrence and nonrecurrence groups were provided.

The exclusion criteria were: non-human study; abstracts, reviews, unpublished reports, overlapped publications, non-English language articles; and AF patients underwent rate control strategies or surgical ablation.

### 2.3. Data extraction

Two reviewers independently extracted data, including publication information (first author name, publication year, and geographic region), study design, characteristics of participants (total number, mean age, gender proportions, AF and ablation type), follow-up duration, recurrence rates, and pre-ablation natriuretic peptide level of recurrence and nonrecurrence groups. Disagreement data were resolved by consensus or adjudication by a third author.

### 2.4. Quality assessment

The Newcastle–Ottawa Scale^[10]^ was used to evaluate the quality of each study. It allocates 0 or 1 point to each numbered item in each category with the exception of comparability where up to 2 points can be assigned. The assessments were done independently by 2 authors. Any disagreements encountered were resolved by discussion. When no consensus could be achieved, a third reviewer was consulted for reconciliation.

### 2.5. Statistical analysis

Standardized mean difference (SMD) was used to present the pooled effect with a 95% confidence interval (CI). The SMD is an estimate of the effect size and can be interpreted as the difference between means of groups divided by the pooled SD. For baseline characteristics represented by median and range/IQR, mean and SD values were estimated using the method described by Luo et al^[11]^ and McGrath et al.^[12]^ Heterogeneity was assessed using the Cochrane *Q* test and *I*^2^ index. When the heterogeneity test was *P* ≥ .05, or *I*^2^ < 50% indicating low statistical heterogeneity, a fixed effect model was used; otherwise, a random effect model was chosen. The source of heterogeneity was analyzed using subgroup analysis. Sensitivity analysis was evaluated by determining whether the remaining results would be markedly affected after removing the relatively low-quality study. Overall effect analysis and subgroup analysis were performed with Review Manager software (RevMan Version 5.4.1, The Nordic Cochrane Center, The Cochrane Collaboration, Copenhagen, Denmark). Publication bias was appraised by visual inspection of funnel plots and Egger test conducted by Stata 15 (Stata Corp., College Station, TX). A trim-and-fill analysis was applied to investigate the potential influence of publication bias. *P* < .05 was considered statistically significant.

### 2.6. Ethics statement

An ethics statement is not applicable because this study is based exclusively on published literature. This study protocol conforms to the provisions of the Helsinki Declaration as revised in 2013.

## 3. Results

### 3.1. Description of included studies

**Figure 1.**
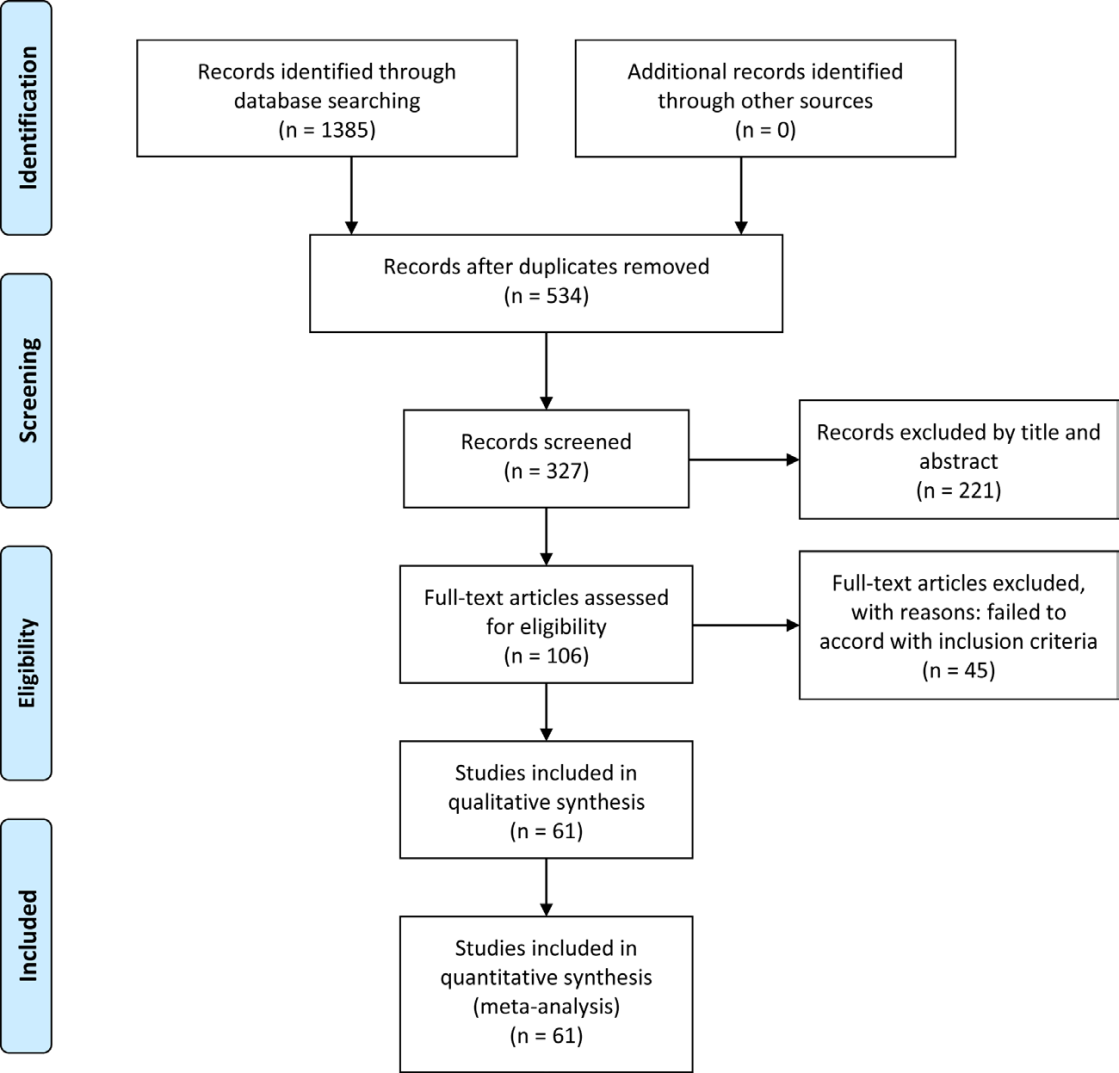
Flow diagram of literature search and study selection.

One thousand three hundred eighty-five studies were acquired after a primary search, and then 851 duplicates were excluded. Three hundred twenty-seven studies underwent the first sift-prescreening by scanning the title and abstracts, among which 221 were excluded. The remaining 106 studies were retrieved for the second selection, and the full texts were read and evaluated carefully. The flow diagram of studies screening was listed in Figure 1. Finally, 61 studies that met the inclusion criteria were included in our meta-analysis. Among them, 8 studies evaluated the association between baseline atrial natriuretic peptide (ANP) levels and AF recurrence after CA, 37 studies evaluated for brain natriuretic peptide (BNP), 25 studies evaluated for N-terminal pro-BNP (NT-proBNP), and 4 studies evaluated for midregional N-terminal pro-ANP (MR-proANP). The demographic and clinical properties of eligible studies are presented in Table 1 and Supplementary Table S1, Supplemental Digital Content, http://links.lww.com/MD/I921. For quality assessment, all included studies were of relatively high quality with the Newcastle–Ottawa Scale scores ranging from 6 to 8 points.

**Table 1:** Characteristics of included studies.

Author	Year	Region	Study design	No. of patients	Mean/Median age (yr)	Gender (M/F)	AF type	Ablation type	Mean/Median follow-up (M)	Recurrence rate (%)	Natriuretic peptide	NOS score
Badoz	2021	France	prospective	105	63	78/27	Paro + pers	RF, CB	12	32.38	MR-proANP	8
Can	2021	Turkey	Prospective	101	60	36/65	Paroxysmal	RF, CB	12	19.8	NT-proBNP	8
Carballo	2018	Switzerland	Prospective	195	57.5	160/35	Paro + pers	RF	6	51.79	NT-proBNP	8
Charitakis	2019	Sweden	Retrospective	189	60.5	134/55	Paro + pers	RF	13.4	62.96	MR-proANP, NT-proBNP	7
Clementy	2018	France	Prospective	75	63	61/14	Persistent	RF	12	33.33	BNP	8
Darkner	2017	Denmark	Prospective	100	60	82/18	Paro + pers	RF	6	44	MR-proANP, NT-proBNP	8
Date	2006	Japan	Prospective	53	53.1	47/6	Paroxysmal	RF	14	39.62	BNP	8
Degener	2011	Germany	Prospective	73	53	56/17	Paro + pers	RF	3	26.03	BNP	7
Deng	2018	China	Retrospective	1410	57.18	960/450	Paro + nonparo	RF, CB	20.7	25.89	BNP	7
Du	2020	China	Retrospective	108	63.1	58/50	Paro + pers	RF	12	22.22	NT-proBNP	8
Elmas	2016	Germany	NA	26	59	21/5	Paroxysmal	CB	6	57.69	MR-proANP, NT-proBNP	6
Fan	2012	China	NA	33	56.4	23/10	Paroxysmal	RF	3	30.3	ANP, NT-proBNP	6
Fiala	2014	Czech	Prospective	159	59	124/36	Persistent	RF	12	18.24	NT-proBNP	8
Giannopoulos	2015	Greece	Retrospective	296	60	207/89	Paroxysmal	RF	13.7	31.42	NT-proBNP	8
Hammache	2021	France	Retrospective	389	58.1	256/133	Paroxysmal	RF	12	32.9	BNP	7
Huang	2014	China	Retrospective	120	50.8	71/49	Paro + pers	RF	12	22.5	BNP	6
Huang	2020	China	Retrospective	422	64	274/148	Paro + pers	RF, CB	12	22.75	BNP	7
Hwang	2009	South Korea	Prospective	73	52	64/9	Paro + pers	RF	3	27.4	NT-proBNP	7
Im	2013	South Korea	NA	499	56.4	367/132	Paro + pers	RF	25.2	24.45	BNP	8
Kimura	2014	Japan	Prospective	44	59	38/6	Paro + pers	RF	9.7	34.09	ANP, BNP	7
Kishima	2018	Japan	Retrospective	106	68	59/47	Paroxysmal	RF	14.6	33.02	BNP	7
Kurosaki	2007	Japan	Retrospective	54	58	46/8	Paro + pers	RF	3	22.22	ANP, BNP	7
Liu	2019	China	Retrospective	67	71.2	46/21	Paro + pers	CB	12	16.42	NT-proBNP	6
Liu	2020	China	Prospective	258	61.0	146/112	Paro + nonparo	NA	13.5	20.16	NT-proBNP	7
Luetkens	2018	Germany	Prospective	61	59.9	40/21	Paro + pers	CB	12	32.79	NT-proBNP	8
Ma	2017	China	Prospective	120	64	72/48	Paro + pers	RF	12	32.5	NT-proBNP	8
Machino-Ohtsuka	2011	Japan	NA	155	61	123/32	Paro + pers	RF	33.8	29.03	BNP	8
Matsumoto	2021	Japan	Retrospective	230	69	158/72	Paro + pers	RF	16.9	20.43	BNP	7
Nakamura	2021	Japan	Prospective	194	64.7	150/44	Paro + nonparo	RF	12	20.1	NT-proBNP	7
Nakanishi	2017	Japan	Retrospective	125	68	80/45	Paro + pers	RF	12.9	32.8	ANP, BNP	7
Nakazawa	2009	Japan	Prospective	51	58	45/6	Paro + pers	RF	6	47.06	ANP, BNP	7
Naruse	2011	Japan	Prospective	221	59	179/42	Paro + pers	RF	31.9	39.37	BNP	8
Nilsson	2009	Denmark	Retrospective	51	NA	NA	Paro + pers	RF	12	56.86	NT-proBNP	7
Ning	2021	China	Retrospective	192	66.64	104/88	NA	RF	>3	35.94	BNP	7
Oka	2020	Japan	Retrospective	292	62	211/81	Paroxysmal	RF	12	45.89	BNP	7
Okada	2021	Japan	Retrospective	217	63	177/40	Paro + pers	RF, CB	35	36.87	BNP	7
Okumura	2011	Japan	Prospective	50	61.3	38/12	Paro + pers	RF	14.0	42	ANP, BNP	8
Parwani	2015	Germany	Prospective	87	62	57/30	Paro + pers	RF	12	28.74	NT-proBNP	8
Pillarisetti	2014	USA	Prospective	88	60	60/28	Paro + pers	RF	12	29.55	BNP	8
Sardana	2016	USA	Prospective	168	60	112/56	Paro + pers	RF, CB	12	46.43	BNP	8
Sardu	2020	Italy	Prospective	27	57.1	17/10	Persistent	RF	12	44.44	BNP	8
Sasaki	2014	Japan	Prospective	60	57.6	49/11	Paro + pers	RF	12.1	48.33	NT-proBNP	8
Shaikh	2015	USA	Retrospective	161	59	113/48	Paro + pers	RF, CB	6	47.83	BNP	7
Shiozawa	2017	Japan	Prospective	77	59	62/15	Paro + pers	RF	19.3	36.36	NT-proBNP	8
Su	2019	China	Prospective	277	65.39	147/130	Paro + pers	RF	14.20	28.16	NT-proBNP	8
Tamura	2019	Japan	Retrospective	227	66	156/71	Paro + pers	RF, CB	15.7	24.67	BNP	7
Tokuda	2011	Japan	Retrospective	224	55.3	187/37	Paroxysmal	RF	37.4	35.71	BNP	7
Uijl	2011	Netherland	Prospective	87	55.0	70/17	Paroxysmal	RF	6.6	24.14	NT-proANP, NT-proBNP	8
Wei	2020	China	Prospective	150	64	85/65	Paro + pers	RF	24	24.67	NT-proBNP	8
Wu	2015	China	Prospective	50	48.9	47/3	Persistent	RF	17	64	BNP	8
Xing	2015	China	NA	36	64.3	21/15	Persistent	RF	3	22.22	BNP	6
Xu	2020	China	Retrospective	233	63.1	195/38	Paroxysmal	RF	3–6	18.03	BNP	6
Yagishita	2017	Japan	NA	34	60.4	27/7	Persistent	RF	6	11.76	BNP	6
Yamada	2006	Japan	NA	66	61	54/12	Paroxysmal	RF	>3	46.97	ANP, BNP	7
Yamaguchi	2017	Japan	NA	100	57.9	88/12	Paro + nonparo	RF	26.2	48	NT-proBNP	6
Yanagisawa	2016	Japan	Retrospective	54	59.8	46/8	Paro + nonparo	RF	6	35.19	BNP	6
Yang	2021	China	NA	215	62.8	180/35	Persistent	RF	3–6	25.58	BNP	6
Yano	2019	Japan	Prospective	168	63.5	105/63	Paro + nonparo	RF	>3	29.76	BNP	7
Yano	2020	Japan	Retrospective	369	70	136/223	Paroxysmal	RF	12	22.49	BNP	7
Yoshida	2015	Japan	NA	137	63	111/26	Paro + pers	RF	27	35.04	ANP, BNP	6
Zou	2013	China	Prospective	76	65.34	39/37	Paro + pers	RF	12	32.89	NT-proBNP	8

AF = atrial fibrillation, ANP = atrial natriuretic peptide, BNP = brain natriuretic peptide, CB = cryoballoon, F = female, M = male, MR-proANP = midregional N-terminal pro-ANP, NA = not available, Nonparo = non-paroxysmal AF, NOS = Newcastle–Ottawa Scale, NT-proBNP = N-terminal pro-brain natriuretic peptide, Paro = paroxysmal AF, Pers = persistent AF, RF = radiofrequency.

### 3.2. Baseline ANP level and the post-ablation AF recurrence

**Figure 2.**
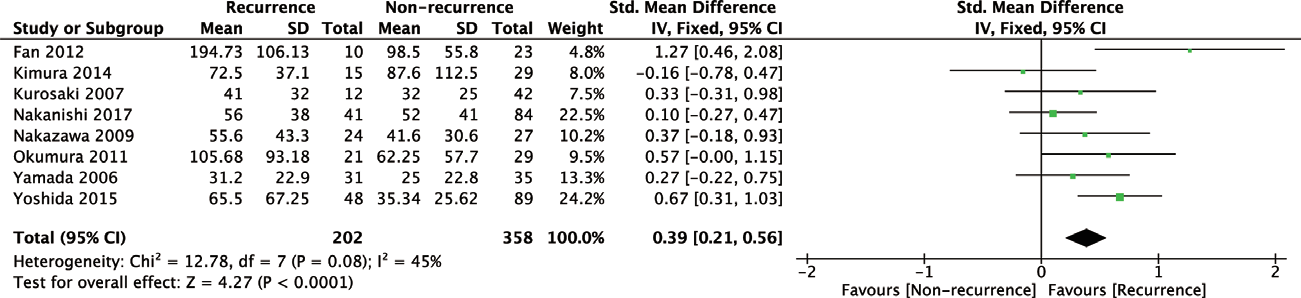
Forrest plot showing the association of ANP with the post-ablation AF recurrence. AF = atrial fibrillation, ANP = atrial natriuretic peptide.

After meta-analysis of 8 related studies, the pooled results indicated that there was a statistically significant association between baseline ANP level and post-ablation AF recurrence (SMD = 0.39, 95% CI: 0.21–0.56, *P* < .0001; Fig. 2). Heterogeneity testing showed that moderate heterogeneity existed with *I*^2^ = 45% (*P* = .08). The heterogeneity was still present after subgroup analysis by follow-up duration, AF type, or sample size (Supplementary Figure S1, Supplemental Digital Content, http://links.lww.com/MD/I922). Since these underlying confounding factors could not explain the heterogeneity, sensitivity analysis was performed by removing 2 relatively low-quality studies to evaluate the stability and reliability of our results. We found that the heterogeneity decreased significantly by excluding 2 relatively low-quality studies (*I*^2^ = 0%), but the overall pooled effects did not change statistically (SMD = 0.23, 95% CI: 0.02–0.44, *P* = .03; Supplementary Figure S2, Supplemental Digital Content, http://links.lww.com/MD/I923), suggesting the result reliable.

### 3.3. Baseline BNP level and the post-ablation AF recurrence

**Figure 3.**
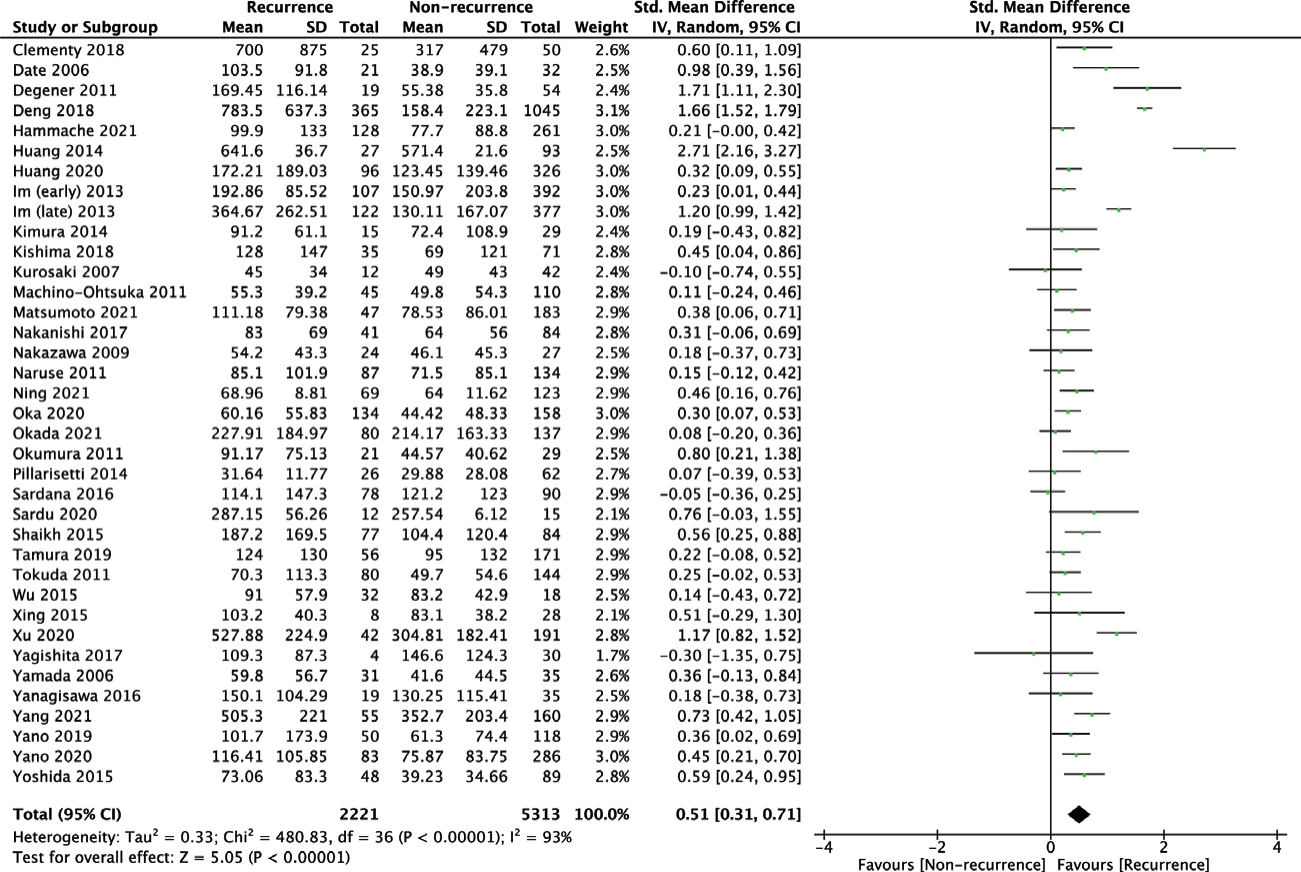
Forrest plot showing the association of BNP with the post-ablation AF recurrence. AF = atrial fibrillation, NT-proBNP = N-terminal pro-brain natriuretic peptide.

Based on the 37 related studies, our meta-analysis showed that the AF recurrence group had a significantly greater baseline BNP level than the nonrecurrence group, and the pooled SMD was 0.51 (95% CI: 0.31–0.71, *P* < .00001; Fig. 3). However, heterogeneity testing revealed a significant heterogeneity (*I*^2^ = 93%). Moderate or high heterogeneity still existed after subgroup analysis by follow-up duration, AF type, sample size, or geographic region (Supplementary Figure S3, Supplemental Digital Content, http://links.lww.com/MD/I924). In sensitivity analysis, the result was not significantly altered after excluding 7 relatively low-quality studies (SMD = 0.44, 95% CI: 0.23–0.66, *P* < .0001; Supplementary Figure S4, Supplemental Digital Content, http://links.lww.com/MD/I925), demonstrating the results were stable.

### 3.4. Baseline NT-proBNP level and the post-ablation AF recurrence

**Figure 4.**
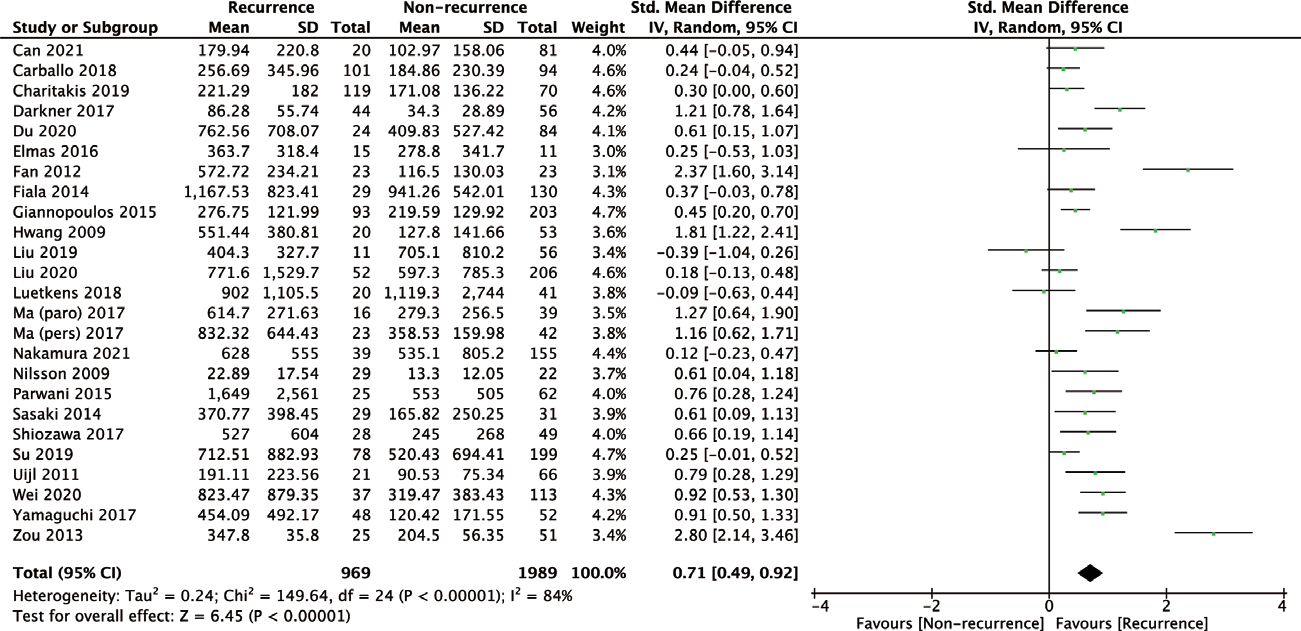
Forrest plot showing the association of NT-proBNP with the post-ablation AF recurrence. AF = atrial fibrillation, NT-proBNP = N-terminal pro-brain natriuretic peptide.

Significant association between pre-ablation baseline NT-proBNP level and post-ablation AF recurrence was found in our meta-analysis of 25 related studies. The pooled SMD was 0.71 (95% CI: 0.49–0.92, *P* < .00001, Fig. 4). Meanwhile, there was a remarkable heterogeneity among the studies with *I*^2^ = 84% (*P* < .00001). After subgroup analysis by follow-up duration, AF type, sample size, or geographic region, moderate or high heterogeneity still existed (Supplementary Figure S5, Supplemental Digital Content, http://links.lww.com/MD/I926). To evaluate the stability and reliability of our results, sensitivity analysis was performed by removing 4 relatively low-quality studies, and our result was not altered (SMD = 0.69, 95% CI: 0.48–0.91, *P* < .00001; Supplementary Figure S6, Supplemental Digital Content, http://links.lww.com/MD/I927).

### 3.5. Baseline MR-proANP level and the post-ablation AF recurrence

**Figure 5.**
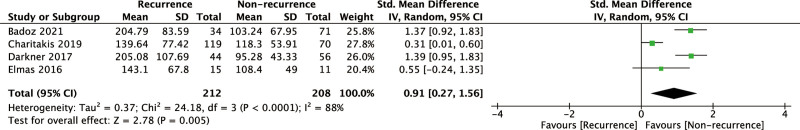
Forrest plot showing the association of MR-proANP with the post-ablation AF recurrence. AF = atrial fibrillation, ANP = atrial natriuretic peptide, MR-proANP = midregional N-terminal pro-ANP.

There were 4 studies that provided data about baseline MR-proANP levels. A statistically significant association was found between baseline MR-proANP level and post-ablation AF recurrence. The pooled SMD was 0.91 (95% CI: 0.27–1.56, *P* = .005, Fig. 5). Meanwhile, there was still a remarkable heterogeneity among the studies with *I*^2^ = 88% (*P* < .0001). In sensitivity analysis, the result was not significantly altered after excluding the relatively low-quality study (SMD = 1.01, 95% CI: 0.23–1.79, *P* = .01; Supplementary Figure S7, Supplemental Digital Content, http://links.lww.com/MD/I928), demonstrating the results were stable.

### 3.6. Publication bias analysis

Publication bias analysis was performed when 10 or more studies were retrieved (BNP and NT-proBNP). The funnel plots of BNP were symmetrical with Egger test *P* = .079, revealing no publication bias was present (Supplementary Figure S8A, Supplemental Digital Content, http://links.lww.com/MD/I929). Nevertheless, the asymmetrical funnel plots and the Egger test for NT-proBNP suggested the presence of publication bias (*P* = .004) (Supplementary Figure S8B, Supplemental Digital Content, http://links.lww.com/MD/I929). However, based on the trim-and-fill analysis, no trimming was performed and data was unchanged (Supplementary Figure S9, Supplemental Digital Content, http://links.lww.com/MD/I930). Combined with the results of the sensitivity analysis, in which the result was not altered, we considered the final results were reliable.

## 4. Discussion

CA, mainly through PVI, is an effective treatment to restore sinus rhythm for patients with AF,^[13]^ but the prognosis after therapy is complex, this includes recurrence or recurrence-free, short-term or long-term recurrence. Predictors for the prognosis are also miscellaneous.^[8,14,15]^ Therefore, identifying and predicting high-risk patients with AF recurrence can help doctors to make an optimized patient selection, inform patients of the risk-benefit ratio, guide surgeons to select the best ablation strategy, and implement individualized treatment strategies. Natriuretic peptides refer to a class of cardiac neurohormones secreted from myocardium cells mostly in response to increased wall tension due to pressure or volume overload.^[16]^ The value of natriuretic peptides as biomarkers useful for cardiovascular diseases was first described in patients with heart failure and continued in those with acute coronary syndrome presentations.^[1]^ Over the past decade, there has also been growing evidence of the use of natriuretic peptides in AF.^[17,18]^ Although the utilization of NPs in heart failure management has been well established, the importance of these biomarkers in relation to AF has not been fully clarified. Consequently, comprehensively analyzing the role of different kinds of natriuretic peptides in AF management may thus facilitate the integration of these widely available biomarkers in clinical applications.

It is worth mentioning that this is one of the few articles to investigate the effect of different natriuretic peptides on the recurrence of AF after ablation with the widest variety of natriuretic peptides. This meta-analysis identified 61 observational studies that investigated the potential association between baseline natriuretic peptides and AF recurrence after CA. The present results suggested that patients with AF recurrence have greater baseline ANP, BNP, NT-proBNP, and MR-proANP levels, indicating a predictive role for natriuretic peptides in the recurrence of AF after CA.

ANP is primarily expressed and stored in the atrium and synthesized as pre-prohormones. The primary stimulus for ANP release is atrial wall stretch resulting from increased intravascular volume.^[19]^ The plasma level of ANP in healthy individuals is approximately 20 pg/mL and is evaluated to be 10- to 100-fold higher in patients with heart failure.^[20]^ Meanwhile, it is also well known that ANP is increased in the setting of atrial tachyarrhythmias, including AF, independent of the left atrium diameter.^[17]^ The elevated concentration of ANP in the peripheral plasma obtained during persistent AF is considered to be caused by the loss of atrial contraction and the rapid ventricular rate, which leads to an increased central volume loading and atrial stretch.^[21]^ In this meta-analysis, 8 studies were pooled and a significant association between baseline ANP level and post-ablation AF recurrence was found. Previous meta-analysis had also revealed the same result.^[6]^

BNP is minimally stored in granules in the ventricles and secreted directly in large bursts following stimulation.^[22]^ The plasma level of BNP in healthy individuals is approximately 3.5 pg/mL and is evaluated to be 100-fold higher in patients with heart failure.^[23]^ Atrial dysrhythmia would also increase BNP secretion. Asynchronous contraction of the atrial myocardium could produce a tethering effect of atrial myocardial fibers that may stimulate the secretion of BNP.^[24]^ Our findings highlight a strong association between elevated BNP and AF recurrence following CA. Another important finding is that BNP levels were affected by follow-up time and region in the subgroup analysis. Patients with short follow-up time (≤3 months) within the cohorts reduced the strength of the association of BNP levels with ablation outcomes. Furthermore, the pooled results for the American region still show no significant association between them (SMD = 0.20, 95% CI: −0.21 to 0.61, *P* = .33), even though Shaikh et al^[25]^ showed that circulating BNP levels were independently associated with late AF recurrence after pulmonary vein isolation.

NT-proBNP, coexisting in circulation with BNP in 1 to 1, is easy to be determined because of its longer half-life (3 to 4 times longer than BNP), higher quantity (16 to 20-fold higher than BNP), and more stable concentration in the blood, which makes NT-proBNP concentrations relatively more stable than BNP over brief time periods.^[26]^ Thus, plasma NT-proBNP level as a cardiac biomarker may be an interesting alternative to BNP.^[27]^ Based on 25 related studies, our meta-analysis shows that the AF recurrence group had a significantly higher pre-ablation level of NT-proBNP. In 2009, Hwang et al demonstrated that NT-proBNP levels were an independent predictor of AF recurrence in a group of patients with paroxysmal AF and persistent AF after multivariate analysis.^[28]^ Besides, NT-proBNP may be a possible biomarker of the hormonal status subsequently reflecting the hormonal remodeling of the atria in patients with AF.^[27]^ Meanwhile, some studies have failed to find a significant association between NT-proBNP and AF recurrence. Giannopolous et al performed a post hoc analysis of a prospective study of hypertensive patients with paroxysmal AF. Baseline NT-proBNP levels were higher in patients with recurrence than in those who remained arrhythmia-free, but the association was rendered non-significant when adjusted for variables.^[29]^

MR-proANP is a stable peptide that results from the cleavage of proANP (pro-ANP)^[30]^ and proANP is stored and released by atrial cardiomyocytes and is cleaved into mature ANP and its N-terminal fragment, NT-proANP.^[31]^ Within this fragment is the midregional section, MR-proANP, which has a longer half-life than mature ANP, making its assessment more reliable.^[32]^ It was previously shown that MR-proANP is elevated in numerous pathologies specifically affecting the left atrium, such as mitral stenosis.^[30,33]^ Meanwhile, MR-proANP is increased in patients with AF, independently of hemodynamic conditions, and also that there is a link between AF burden and MR-proANP levels.^[34]^ Other studies have also reported that CA for AF significantly reduced plasma concentrations of ANP and MR-proANP in the long term.^[35]^ In our meta-analysis, 4 studies were pooled and showed a statistically significant association between baseline MR-proANP level and post-ablation AF recurrence.

The explanation for the predictive role of natriuretic peptides in the recurrence of AF may be as follows: Firstly, it has been reported that elevated levels of natriuretic peptides are associated with an increase in left atrial size, which may increase the risk of recurrence of AF due to atrial fibrosis and remodeling after ablation.^[36]^ Secondly, natriuretic peptide is recognized as a diagnostic marker of heart failure. The increase of natriuretic peptide levels before ablation may reflect cardiac dysfunction, which may cause AF through intracellular calcium overload, worsening myocardial fibrosis, decreased conduction velocity, and increased dispersity of the refractory period.^[37]^

## 5. Limitation

Undoubtedly, there were several potential limitations in this meta-analysis. First, the retrieved studies in our meta-analysis were observational studies rather than randomized control trials, in which comparability between groups was not easy to be controlled. Some of them had a relatively small sample size. Second, Moderate or high heterogeneity existed among studies that were utilized for assessments. The contributing factors to heterogeneity include variation in follow-up time (from 3 to 63 months), sample size (from 26 to 1410), and AF type. Third, our inclusion criteria focused on studies in English, which led to selection or allocation biases, and affected the results of our meta-analysis. Fourth, we did not assess the prognostic value of baseline natriuretic peptides level by continuous variable due to insufficient such data. Finally, publication bias was found in the studies pooling NT-proBNP outcomes. However, the “trim-and-fill” analysis indicated that the predictive value of NT-proBNP level had not changed obviously after adjustment of publication bias.

## 6. Conclusion

In conclusion, our meta-analysis indicated that increased baseline natriuretic peptide levels, including ANP, BNP, NT-proBNP, and MR-proANP, are associated with a higher risk of AF recurrence after CA. Measurement of these baseline levels should be recommended among AF patients before CA. Considering the limitations described above, further well-designed, larger, and long-term studies are needed to elucidate the predictive value of baseline natriuretic peptides in AF patients undergoing CA.

## Author contributions

**Conceptualization:** Yujing Yuan, Boyuan Nie.

**Data curation:** Boyuan Nie, Li Li.

**Formal analysis:** Yujing Yuan, Boyuan Nie, Li Li.

**Investigation:** Caixia Guo.

**Methodology:** Yujing Yuan, Boyuan Nie, Binbin Gao.

**Resources:** Caixia Guo.

**Software:** Binbin Gao.

**Supervision:** Boyuan Nie, Binbin Gao, Caixia Guo.

**Writing – original draft:** Yujing Yuan.

**Writing – review & editing:** Yujing Yuan, Binbin Gao.

## Supplementary Material




















