# Supplementary material for: Natriuretic peptides as predictors for atrial fibrillation recurrence after catheter ablation: A meta-analysis
Source: Medicine (Baltimore). 2023 May 12;102(19):e33704. doi: 10.1097/MD.0000000000033704 (PMC10174372; doi:10.1097/MD.0000000000033704)
Supplement: Supplementary file 1 [file medi-102-e33704-s001.pdf]

Supplementary Table S1 References for included studies

Author	Year	Reference
**Badoz**	2021	Badoz M, Serzian G, Favoulet B, et al. Impact of Midregional N-Terminal Pro-Atrial Natriuretic Peptide and Soluble Suppression of Tumorigenicity 2 Levels on Heart Rhythm in Patients Treated With Catheter Ablation for Atrial Fibrillation: The Biorhythm Study. Journal of the American Heart Association. 2021;10(13):e020917.
**Can**	2021	Can V, Cakmak HA, Vatansever F, et al. Assessment of the relationship between semaphorin4D level and recurrence after catheter ablation in paroxysmal atrial fibrillation. Biomarkers : biochemical indicators of exposure, response, and susceptibility to chemicals. 2021;26(5):468-476.
**Carballo**	2018	Carballo D, Noble S, Carballo S, et al. Biomarkers and arrhythmia recurrence following radiofrequency ablation of atrial fibrillation. The Journal of international medical research. 2018;46(12):5183-5194.
**Charitakis**	2019	Charitakis E, Karlsson LO, Papageorgiou JM, Walfridsson U, Carlhäll CJ. Echocardiographic and Biochemical Factors Predicting Arrhythmia Recurrence After Catheter Ablation of Atrial Fibrillation-An Observational Study. Frontiers in physiology. 2019;10:1215.
**Clementy**	2018	Clementy N, Garcia B, André C, et al. Galectin-3 level predicts response to ablation and outcomes in patients with persistent atrial fibrillation and systolic heart failure. PloS one. 2018;13(8):e0201517.
**Darkner**	2017	Darkner S, Goetze JP, Chen X, Henningsen K, Pehrson S, Svendsen JH. Natriuretic Propeptides as Markers of Atrial Fibrillation Burden and Recurrence (from the AMIO-CAT Trial). The American journal of cardiology. 2017;120(8):1309-1315.
**Date**	2006	Date T, Yamane T, Inada K, et al. Plasma brain natriuretic peptide concentrations in patients undergoing pulmonary vein isolation. Heart (British Cardiac Society). 2006;92(11):1623-1627.
**Degener**	2011	Degener S, Pattberg SV, Feuersenger H, et al. Predictive value of B-type natriuretic peptide levels in patients with paroxysmal and persistent atrial fibrillation undergoing pulmonary vein isolation. Journal of interventional cardiac electrophysiology : an international journal of arrhythmias and pacing. 2011;30(3):217-225.
**Deng**	2018	Deng H, Shantsila A, Guo P, et al. Multiple biomarkers and arrhythmia outcome following catheter ablation of atrial fibrillation: The Guangzhou Atrial Fibrillation Project. Journal of arrhythmia. 2018;34(6):617-625.
**Du**	2020	Du W, Dai M, Wang M, et al. Large left atrial appendage predicts the ablation outcome in hypertensive patients with atrial fibrillation. Journal of electrocardiology. 2020;63:139-144.
**Elmas**	2016	Elmas E, Tülümen E, Liebe V, et al. Mid-regional pro-adrenomedullin and N-terminal pro B-type natriuretic peptide predict the recurrence of atrial fibrillation after cryoballoon pulmonary vein isolation. International journal of cardiology. 2016;203:369-371.
**Fan**	2012	Fan J, Cao H, Su L, et al. NT-proBNP, but not ANP and C-reactive protein, is predictive of paroxysmal atrial fibrillation in patients undergoing pulmonary vein isolation. Journal of interventional cardiac electrophysiology : an international journal of arrhythmias and pacing. 2012;33(1):93-100.
**Fiala**	2014	Fiala M, Wichterle D, Bulková V, et al. A prospective evaluation of haemodynamics, functional status, and quality of life after radiofrequency catheter ablation of long-standing persistent atrial fibrillation. Europace : European pacing, arrhythmias, and cardiac electrophysiology : journal of the working groups on cardiac pacing, arrhythmias, and cardiac cellular

		electrophysiology of the European Society of Cardiology. 2014;16(1):15-25.
**Giannopoulos**	2015	Giannopoulos G, Kossyvakis C, Angelidis C, et al. Amino-terminal B-natriuretic peptide levels and postablation recurrence in hypertensive patients with paroxysmal atrial fibrillation. Heart rhythm. 2015;12(7):1470-1475.
**Hammache**	2021	Hammache N, Pegorer-Sfes H, Benali K, et al. Is There an Association between Epicardial Adipose Tissue and Outcomes after Paroxysmal Atrial Fibrillation Catheter Ablation? Journal of clinical medicine. 2021;10(14).
**Huang**	2014	Huang Q, Yuan Y, Qiu C, et al. Effect of catheter radiofrequency ablation on C-reactive protein, brain natriuretic peptide and echocardiograph in patients with persistent and permanent atrial fibrillation. Chinese medical journal. 2014;127(4):623-626.
**Huang**	2020	Huang Z, Liang X, Wang W, et al. Relationship between plasma cancer antigen (CA)-125 level and one-year recurrence of atrial fibrillation after catheter ablation. Clinica chimica acta; international journal of clinical chemistry. 2020;502:201-206.
**Hwang**	2009	Hwang HJ, Son JW, Nam BH, et al. Incremental predictive value of pre-procedural N-terminal pro-B-type natriuretic peptide for short-term recurrence in atrial fibrillation ablation. Clinical research in cardiology : official journal of the German Cardiac Society. 2009;98(4):213-218.
**Im**	2013	Im SI, Shin SY, Na JO, et al. Usefulness of neutrophil/lymphocyte ratio in predicting early recurrence after radiofrequency catheter ablation in patients with atrial fibrillation. International journal of cardiology. 2013;168(4):4398-4400.
**Kimura**	2014	Kimura T, Takatsuki S, Inagawa K, et al. Serum inflammation markers predicting successful initial catheter ablation for atrial fibrillation. Heart, lung & circulation. 2014;23(7):636-643.
**Kishima**	2018	Kishima H, Mine T, Takahashi S, Ashida K, Ishihara M, Masuyama T. Left atrial ejection force predicts the outcome after catheter ablation for paroxysmal atrial fibrillation. Journal of cardiovascular electrophysiology. 2018;29(2):264-271.
**Kurosaki**	2007	Kurosaki K, Tada H, Hashimoto T, et al. Plasma natriuretic peptide concentrations as a predictor for successful catheter ablation in patients with drug-refractory atrial fibrillation. Circulation journal : official journal of the Japanese Circulation Society. 2007;71(3):313-320.
**Liu**	2019	Liu L, Zhao D, Zhang J, et al. Impact of Stable Coronary Artery Disease on the Efficacy of Cryoballoon Ablation for the Atrial Fibrillation. The American journal of the medical sciences. 2019;358(3):204-211.
**Liu**	2020	Liu H, Wang K, Lin Y, et al. Role of sST2 in predicting recurrence of atrial fibrillation after radiofrequency catheter ablation. Pacing and clinical electrophysiology : PACE. 2020;43(11):1235-1241.
**Luetkens**	2018	Luetkens JA, Wolpers AC, Beiert T, et al. Cardiac magnetic resonance using late gadolinium enhancement and atrial T1 mapping predicts poor outcome in patients with atrial fibrillation after catheter ablation therapy. Scientific reports. 2018;8(1):13618.
**Ma**	2017	Ma XX, Zhang YL, Hu B, et al. Association between left atrial appendage emptying velocity, N-terminal plasma brain natriuretic peptide levels, and recurrence of atrial fibrillation after catheter ablation. Journal of interventional cardiac electrophysiology : an international journal of arrhythmias and pacing. 2017;48(3):343-350.

**Machino-Ohtsuka**	2011	Machino-Ohtsuka T, Seo Y, Tada H, et al. Left atrial stiffness relates to left ventricular diastolic dysfunction and recurrence after pulmonary vein isolation for atrial fibrillation. Journal of cardiovascular electrophysiology. 2011;22(9):999-1006.
**Matsumoto**	2021	Matsumoto S, Matsunaga-Lee Y, Ishimi M, et al. Clinical Significance of B-Type Natriuretic Peptide Levels at 3 Months after Atrial Fibrillation Ablation. Diseases (Basel, Switzerland). 2021;9(3).
**Nakamura**	2021	Nakamura K, Takagi T, Kogame N, et al. Impact of atrial mitral and tricuspid regurgitation on atrial fibrillation recurrence after ablation. Journal of electrocardiology. 2021;66:114-121.
**Nakanishi**	2017	Nakanishi K, Fukuda S, Yamashita H, et al. High-sensitive cardiac troponin T as a novel predictor for recurrence of atrial fibrillation after radiofrequency catheter ablation. Europace : European pacing, arrhythmias, and cardiac electrophysiology : journal of the working groups on cardiac pacing, arrhythmias, and cardiac cellular electrophysiology of the European Society of Cardiology. 2017;19(12):1951-1957.
**Nakazawa**	2009	Nakazawa Y, Ashihara T, Tsutamoto T, Ito M, Horie M. Endothelin-1 as a predictor of atrial fibrillation recurrence after pulmonary vein isolation. Heart rhythm. 2009;6(6):725-730.
**Naruse**	2011	Naruse Y, Tada H, Sekiguchi Y, et al. Concomitant chronic kidney disease increases the recurrence of atrial fibrillation after catheter ablation of atrial fibrillation: a mid-term follow-up. Heart rhythm. 2011;8(3):335-341.
**Nilsson**	2009	Nilsson B, Goetze JP, Chen X, Pehrson S, Svendsen JH. Increased NT-pro-B-type natriuretic peptide independently predicts outcome following catheter ablation of atrial fibrillation. Scandinavian journal of clinical and laboratory investigation. 2009;69(8):843-850.
**Ning**	2021	Ning Z, Li X, Zhu X, Luo J, Wu Y. Relationship between serum angiopoietin-like 4 levels and recurrence of atrial fibrillation. The Journal of international medical research. 2021;49(2):300060520988393.
**Oka**	2020	Oka T, Tanaka K, Ninomiya Y, et al. Impact of baseline left atrial function on long-term outcome after catheter ablation for paroxysmal atrial fibrillation. Journal of cardiology. 2020;75(4):352-359.
**Okada**	2021	Okada M, Tanaka N, Tanaka K, et al. Usefulness of Post-Procedural Plasma Brain Natriuretic Peptide Levels to Predict Recurrence After Catheter Ablation of Atrial Fibrillation in Patients With Left Ventricular Systolic Dysfunction. The American journal of cardiology. 2021;144:67-76.
**Okumura**	2011	Okumura Y, Watanabe I, Nakai T, et al. Impact of biomarkers of inflammation and extracellular matrix turnover on the outcome of atrial fibrillation ablation: importance of matrix metalloproteinase-2 as a predictor of atrial fibrillation recurrence. Journal of cardiovascular electrophysiology. 2011;22(9):987-993.
**Parwani**	2015	Parwani AS, von Haehling S, Kolodziejski AI, et al. Mid-regional proadrenomedullin levels predict recurrence of atrial fibrillation after catheter ablation. International journal of cardiology. 2015;180:129-133.
**Pillarisetti**	2014	Pillarisetti J, Reddy N, Biria M, et al. Elevated brain natriuretic peptide level in patients undergoing atrial fibrillation ablation: is it a predictor of failed ablation or a mere function of atrial rhythm and rate at a point in time? Journal of interventional cardiac electrophysiology : an international journal of arrhythmias and pacing. 2014;40(2):161-168.
**Sardana**	2016	Sardana M, Ogunsua AA, Spring M, et al. Association of Left Atrial Function Index With Late Atrial Fibrillation Recurrence after Catheter Ablation. Journal of cardiovascular

		electrophysiology. 2016;27(12):1411-1419.
**Sardu**	2020	Sardu C, Santulli G, Guerra G, et al. Modulation of SERCA in Patients with Persistent Atrial Fibrillation Treated by Epicardial Thoracoscopic Ablation: The CAMAF Study. Journal of clinical medicine. 2020;9(2).
**Sasaki**	2014	Sasaki N, Okumura Y, Watanabe I, et al. Increased levels of inflammatory and extracellular matrix turnover biomarkers persist despite reverse atrial structural remodeling during the first year after atrial fibrillation ablation. Journal of interventional cardiac electrophysiology : an international journal of arrhythmias and pacing. 2014;39(3):241-249.
**Shaikh**	2015	Shaikh AY, Esa N, Martin-Doyle W, et al. Addition of B-Type Natriuretic Peptide to Existing Clinical Risk Scores Enhances Identification of Patients at Risk for Atrial Fibrillation Recurrence After Pulmonary Vein Isolation. Critical pathways in cardiology. 2015;14(4):157-165.
**Shiozawa**	2017	Shiozawa T, Shimada K, Sekita G, et al. Left Atrial Appendage Volume and Plasma Docosahexaenoic Acid Levels Are Associated With Atrial Fibrillation Recurrence After Catheter Ablation. Cardiology research. 2017;8(3):96-104.
**Su**	2019	Su C, Liu Z, Gao Y, et al. Study on the relationship between telomere length changes and recurrence of atrial fibrillation after radiofrequency catheter ablation. Journal of cardiovascular electrophysiology. 2019;30(7):1117-1124.
**Tamura**	2019	Tamura S, Doi A, Matsuo M, et al. Prognostic value of high-sensitive troponin T for predicting cardiovascular events after atrial fibrillation ablation. Journal of cardiovascular electrophysiology. 2019;30(9):1475-1482.
**Tokuda**	2011	Tokuda M, Yamane T, Matsuo S, et al. Relationship between renal function and the risk of recurrent atrial fibrillation following catheter ablation. Heart (British Cardiac Society). 2011;97(2):137-142.
**Uijl**	2011	den Uijl DW, Delgado V, Tops LF, et al. Natriuretic peptide levels predict recurrence of atrial fibrillation after radiofrequency catheter ablation. American heart journal. 2011;161(1):197-203.
**Wei**	2020	Wei Y, Liu S, Yu H, et al. The Predictive Value of Growth Differentiation Factor-15 in Recurrence of Atrial Fibrillation after Catheter Ablation. Mediators of inflammation. 2020;2020:8360936.
**Wu**	2015	Wu XY, Li SN, Wen SN, et al. Plasma galectin-3 predicts clinical outcomes after catheter ablation in persistent atrial fibrillation patients without structural heart disease. Europace : European pacing, arrhythmias, and cardiac electrophysiology : journal of the working groups on cardiac pacing, arrhythmias, and cardiac cellular electrophysiology of the European Society of Cardiology. 2015;17(10):1541-1547.
**Xing**	2015	Xing SY, Wang HL, Dong PS, et al. Clinical significance and levels of blood brain natriuretic peptides in patients with persistent atrial fibrillation before and after catheter ablation. Genetics and molecular research : GMR. 2015;14(2):6953-6959.
**Xu**	2020	Xu M, Liu F, Ge ZX, Li JM, Xie X, Yang JH. Functional studies of left atrium and BNP in patients with paroxysmal atrial fibrillation and the prediction of recurrence after CPVA. European review for medical and pharmacological sciences. 2020;24(9):4997-5007.

**Yagishita**	2017	Yagishita A, Yamauchi Y, Sato H, et al. Improvement in the Quality of Life and Exercise Performance in Relation to the Plasma B-Type Natriuretic Peptide Level After Catheter Ablation in Patients With Asymptomatic Persistent Atrial Fibrillation. Circulation journal : official journal of the Japanese Circulation Society. 2017;81(4):444-449.
**Yamada**	2006	Yamada T, Murakami Y, Okada T, et al. Plasma atrial natriuretic Peptide and brain natriuretic Peptide levels after radiofrequency catheter ablation of atrial fibrillation. The American journal of cardiology. 2006;97(12):1741-1744.
**Yamaguchi**	2017	Yamaguchi N, Okumura Y, Watanabe I, et al. Clinical implications of serum adiponectin on progression of atrial fibrillation. Journal of arrhythmia. 2017;33(6):608-612.
**Yanagisawa**	2016	Yanagisawa S, Inden Y, Kato H, et al. Decrease in B-Type Natriuretic Peptide Levels and Successful Catheter Ablation for Atrial Fibrillation in Patients with Heart Failure. Pacing and clinical electrophysiology : PACE. 2016;39(3):225-234.
**Yang**	2021	Yang Z, Xu M, Zhang C, et al. A predictive model using left atrial function and B-type natriuretic peptide level in predicting the recurrence of early persistent atrial fibrillation after radiofrequency ablation. Clinical cardiology. 2021;44(3):407-414.
**Yano**	2019	Yano M, Egami Y, Ukita K, et al. Atrial fibrillation type modulates the clinical predictive value of neutrophil-to-lymphocyte ratio for atrial fibrillation recurrence after catheter ablation. International journal of cardiology. Heart & vasculature. 2020;31:100664.
**Yano**	2020	Yano M, Egami Y, Yanagawa K, et al. Predictors of recurrence after pulmonary vein isolation in patients with normal left atrial diameter. Journal of arrhythmia. 2020;36(1):75-81.
**Yoshida**	2015	Yoshida K, Kaneshiro T, Ito Y, et al. Elevated plasma norepinephrine level and sick sinus syndrome in patients with lone atrial fibrillation. Heart (British Cardiac Society). 2015;101(14):1133-1138.
**Zou**	2013	Zou C, Zhang Z, Zhao W, et al. Predictive value of pre-procedural autoantibodies against M2-muscarinic acetylcholine receptor for recurrence of atrial fibrillation one year after radiofrequency catheter ablation. Journal of translational medicine. 2013;11:7.
