# Supplementary material for: Natriuretic peptides as predictors for atrial fibrillation recurrence after catheter ablation: A meta-analysis
Source: Medicine (Baltimore). 2023 May 12;102(19):e33704. doi: 10.1097/MD.0000000000033704 (PMC10174372; doi:10.1097/MD.0000000000033704)

Figure S1 Subgroup analysis of the association of ANP with the post-ablation AF recurrence. **A** follow-up duration; **B** AF type (paro+pers: both paroxysmal and AF patients); **C** sample size.

A

Study or Subgroup	Recurrence Mean	Recurrence SD	Recurrence Total	Non-recurrence Mean	Non-recurrence SD	Non-recurrence Total	Weight	Std. Mean Difference IV, Fixed, 95% CI
**1.2.1 ≤3 months**								
Fan 2012	194.73	106.13	10	98.5	55.8	23	4.8%	1.27 [0.46, 2.08]
Kurosaki 2007	41	32	12	32	25	42	7.5%	0.33 [−0.31, 0.98]
**Subtotal (95% CI)**			**22**			**65**	**12.3%**	**0.70 [0.19, 1.20]**
Heterogeneity: Chi² = 3.13, df = 1 (P = 0.08); I² = 68%								
Test for overall effect: Z = 2.70 (P = 0.007)								
**1.2.2 3~12 months**								
Kimura 2014	72.5	37.1	15	87.6	112.5	29	8.0%	−0.16 [−0.78, 0.47]
Nakazawa 2009	55.6	43.3	24	41.6	30.6	27	10.2%	0.37 [−0.18, 0.93]
Yamada 2006	31.2	22.9	31	25	22.8	35	13.3%	0.27 [−0.22, 0.75]
**Subtotal (95% CI)**			**70**			**91**	**31.5%**	**0.19 [−0.12, 0.51]**
Heterogeneity: Chi² = 1.70, df = 2 (P = 0.43); I² = 0%								
Test for overall effect: Z = 1.20 (P = 0.23)								
**1.2.3 > 12 months**								
Nakanishi 2017	56	38	41	52	41	84	22.5%	0.10 [−0.27, 0.47]
Okumura 2011	105.68	93.18	21	62.25	57.7	29	9.5%	0.57 [−0.00, 1.15]
Yoshida 2015	65.5	67.25	48	35.34	25.62	89	24.2%	0.67 [0.31, 1.03]
**Subtotal (95% CI)**			**110**			**202**	**56.2%**	**0.43 [0.19, 0.66]**
Heterogeneity: Chi² = 4.96, df = 2 (P = 0.08); I² = 60%								
Test for overall effect: Z = 3.53 (P = 0.0004)								
**Total (95% CI)**			**202**			**358**	**100.0%**	**0.39 [0.21, 0.56]**
Heterogeneity: Chi² = 12.78, df = 7 (P = 0.08); I² = 45%								
Test for overall effect: Z = 4.27 (P < 0.0001)								
Test for subgroup differences: Chi² = 2.99, df = 2 (P = 0.22), I² = 33.1%								

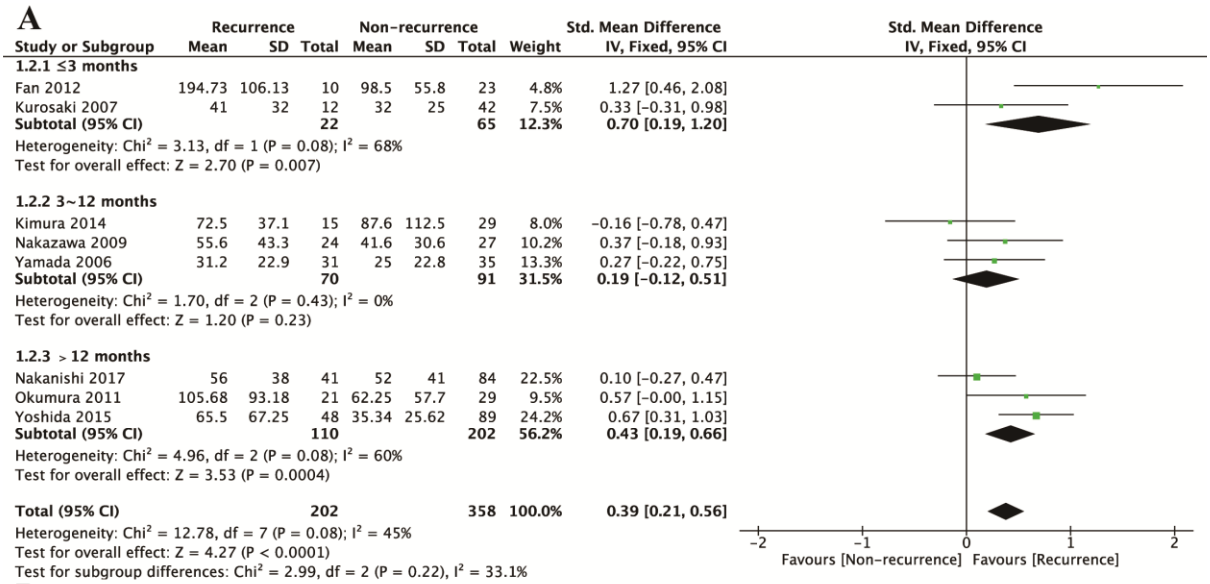

B

Study or Subgroup	Experimental Mean	Experimental SD	Experimental Total	Control Mean	Control SD	Control Total	Weight	Std. Mean Difference IV, Fixed, 95% CI
**1.3.1 paroxysmal**								
Fan 2012	194.73	106.13	10	98.5	55.8	23	4.8%	1.27 [0.46, 2.08]
Yamada 2006	31.2	22.9	31	25	22.8	35	13.3%	0.27 [−0.22, 0.75]
**Subtotal (95% CI)**			**41**			**58**	**18.1%**	**0.53 [0.12, 0.95]**
Heterogeneity: Chi² = 4.30, df = 1 (P = 0.04); I² = 77%								
Test for overall effect: Z = 2.50 (P = 0.01)								
**1.3.2 paro+pers**								
Kimura 2014	72.5	37.1	15	87.6	112.5	29	8.0%	−0.16 [−0.78, 0.47]
Kurosaki 2007	41	32	12	32	25	42	7.5%	0.33 [−0.31, 0.98]
Nakanishi 2017	56	38	41	52	41	84	22.5%	0.10 [−0.27, 0.47]
Nakazawa 2009	55.6	43.3	24	41.6	30.6	27	10.2%	0.37 [−0.18, 0.93]
Okumura 2011	105.68	93.18	21	62.25	57.7	29	9.5%	0.57 [−0.00, 1.15]
Yoshida 2015	65.5	67.25	48	35.34	25.62	89	24.2%	0.67 [0.31, 1.03]
**Subtotal (95% CI)**			**161**			**300**	**81.9%**	**0.35 [0.16, 0.55]**
Heterogeneity: Chi² = 7.89, df = 5 (P = 0.16); I² = 37%								
Test for overall effect: Z = 3.54 (P = 0.0004)								
**Total (95% CI)**			**202**			**358**	**100.0%**	**0.39 [0.21, 0.56]**
Heterogeneity: Chi² = 12.78, df = 7 (P = 0.08); I² = 45%								
Test for overall effect: Z = 4.27 (P < 0.0001)								
Test for subgroup differences: Chi² = 0.58, df = 1 (P = 0.45), I² = 0%								

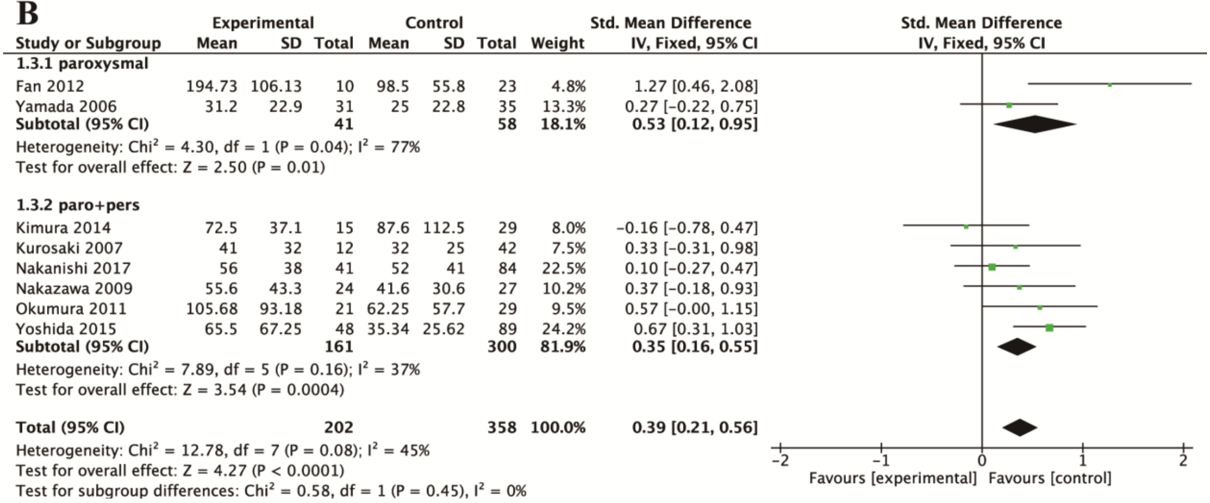

C

Study or Subgroup	Recurrence Mean	Recurrence SD	Recurrence Total	Non-recurrence Mean	Non-recurrence SD	Non-recurrence Total	Weight	Std. Mean Difference IV, Fixed, 95% CI
**1.4.1 ≤100**								
Fan 2012	194.73	106.13	10	98.5	55.8	23	4.8%	1.27 [0.46, 2.08]
Kimura 2014	72.5	37.1	15	87.6	112.5	29	8.0%	−0.16 [−0.78, 0.47]
Kurosaki 2007	41	32	12	32	25	42	7.5%	0.33 [−0.31, 0.98]
Nakazawa 2009	55.6	43.3	24	41.6	30.6	27	10.2%	0.37 [−0.18, 0.93]
Okumura 2011	105.68	93.18	21	62.25	57.7	29	9.5%	0.57 [−0.00, 1.15]
Yamada 2006	31.2	22.9	31	25	22.8	35	13.3%	0.27 [−0.22, 0.75]
**Subtotal (95% CI)**			**113**			**185**	**53.4%**	**0.38 [0.13, 0.62]**
Heterogeneity: Chi² = 8.11, df = 5 (P = 0.15); I² = 38%								
Test for overall effect: Z = 3.05 (P = 0.002)								
**1.4.2 > 100**								
Nakanishi 2017	56	38	41	52	41	84	22.5%	0.10 [−0.27, 0.47]
Yoshida 2015	65.5	67.25	48	35.34	25.62	89	24.2%	0.67 [0.31, 1.03]
**Subtotal (95% CI)**			**89**			**173**	**46.6%**	**0.40 [0.14, 0.65]**
Heterogeneity: Chi² = 4.65, df = 1 (P = 0.03); I² = 79%								
Test for overall effect: Z = 2.99 (P = 0.003)								
**Total (95% CI)**			**202**			**358**	**100.0%**	**0.39 [0.21, 0.56]**
Heterogeneity: Chi² = 12.78, df = 7 (P = 0.08); I² = 45%								
Test for overall effect: Z = 4.27 (P < 0.0001)								
Test for subgroup differences: Chi² = 0.01, df = 1 (P = 0.92), I² = 0%								

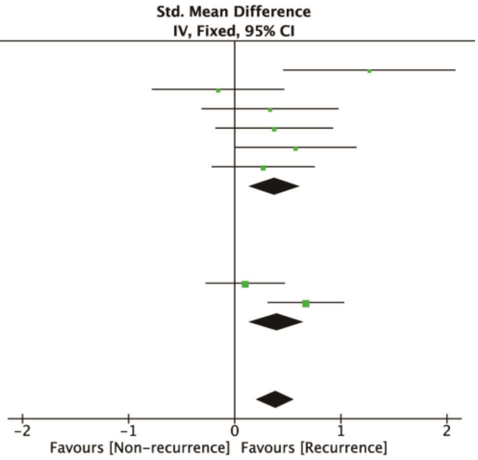

Supplement: Supplementary file 2 [file medi-102-e33704-s002.pdf]
