# Supplementary figures and images for: Natriuretic peptides as predictors for atrial fibrillation recurrence after catheter ablation: A meta-analysis
Source: Medicine (Baltimore). 2023 May 12;102(19):e33704. doi: 10.1097/MD.0000000000033704 (PMC10174372; doi:10.1097/MD.0000000000033704)

**Figure S2** Sensitive analysis of the association of ANP with the post-ablation AF recurrence.

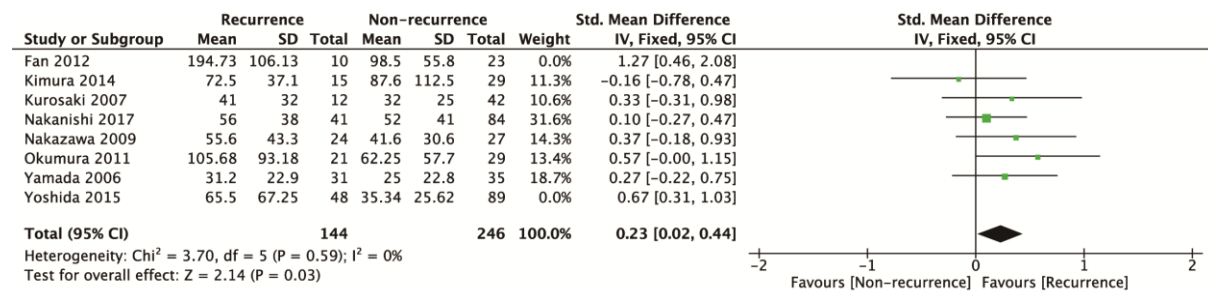

Supplement: Supplementary file 3 [file medi-102-e33704-s003.pdf]

**Figure S4** Sensitive analysis of the association of BNP with the post-ablation AF recurrence.

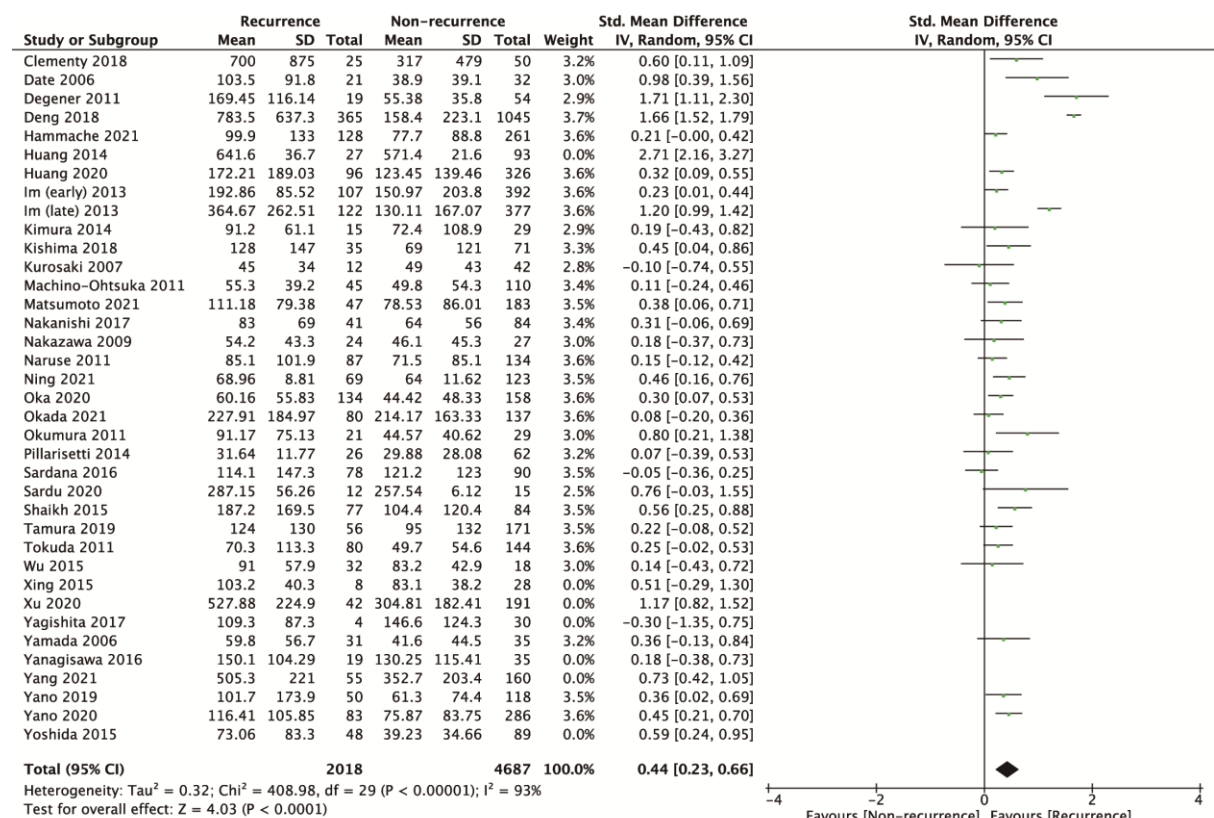

Supplement: Supplementary file 5 [file medi-102-e33704-s005.pdf]

**Figure S8** Funnel plot analysis to detect publication bias. **A** BNP; **B** NT-proBNP.

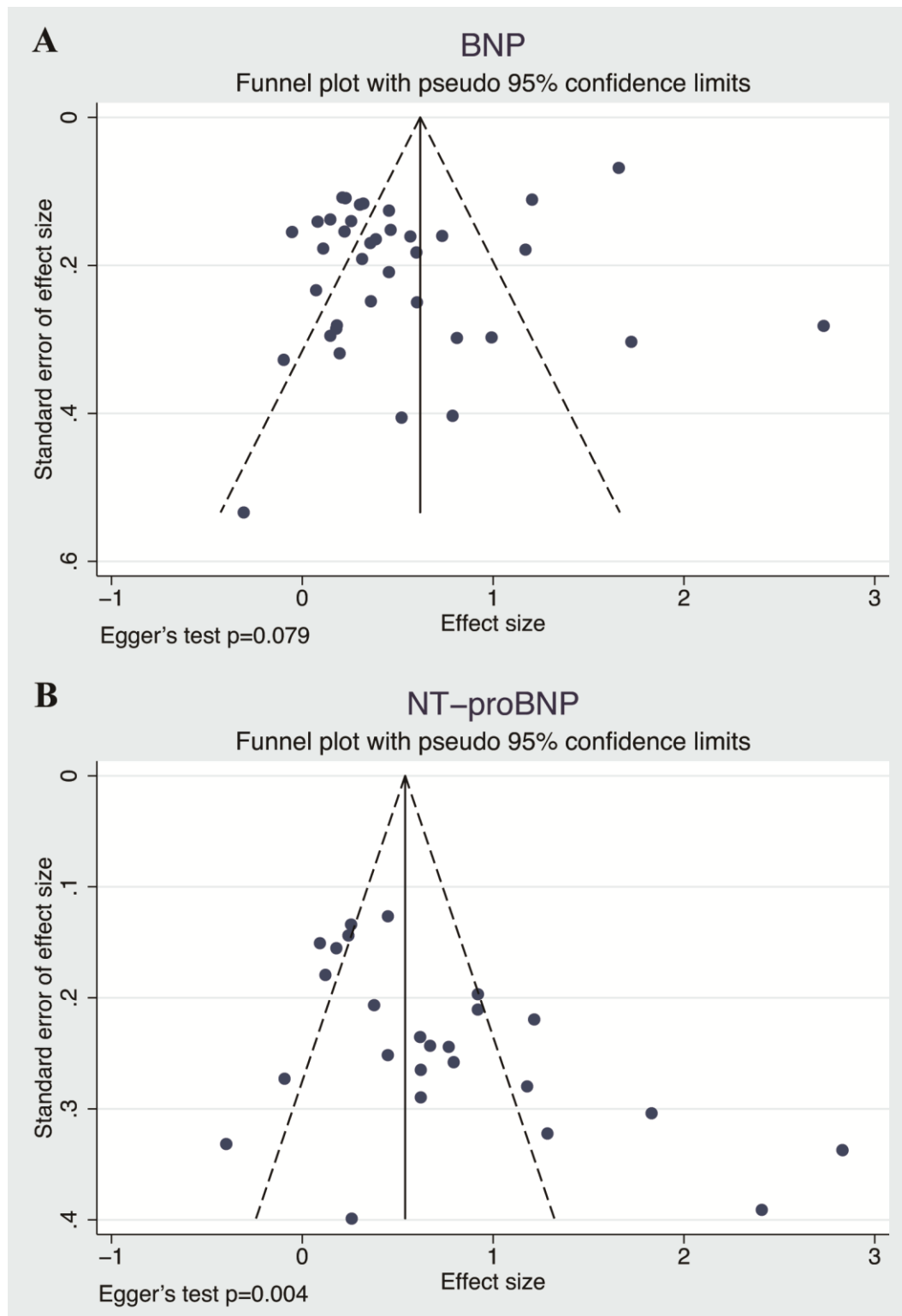

Supplement: Supplementary file 9 [file medi-102-e33704-s009.pdf]
