# Supplementary material for: Natriuretic peptides as predictors for atrial fibrillation recurrence after catheter ablation: A meta-analysis
Source: Medicine (Baltimore). 2023 May 12;102(19):e33704. doi: 10.1097/MD.0000000000033704 (PMC10174372; doi:10.1097/MD.0000000000033704)

Figure S5 Subgroup analysis of the association of NT-proBNP with the post-ablation AF recurrence. **A** follow-up duration; **B** AF type (paro+pers: both paroxysmal and AF patients); **C** sample size; **D** geographic region.

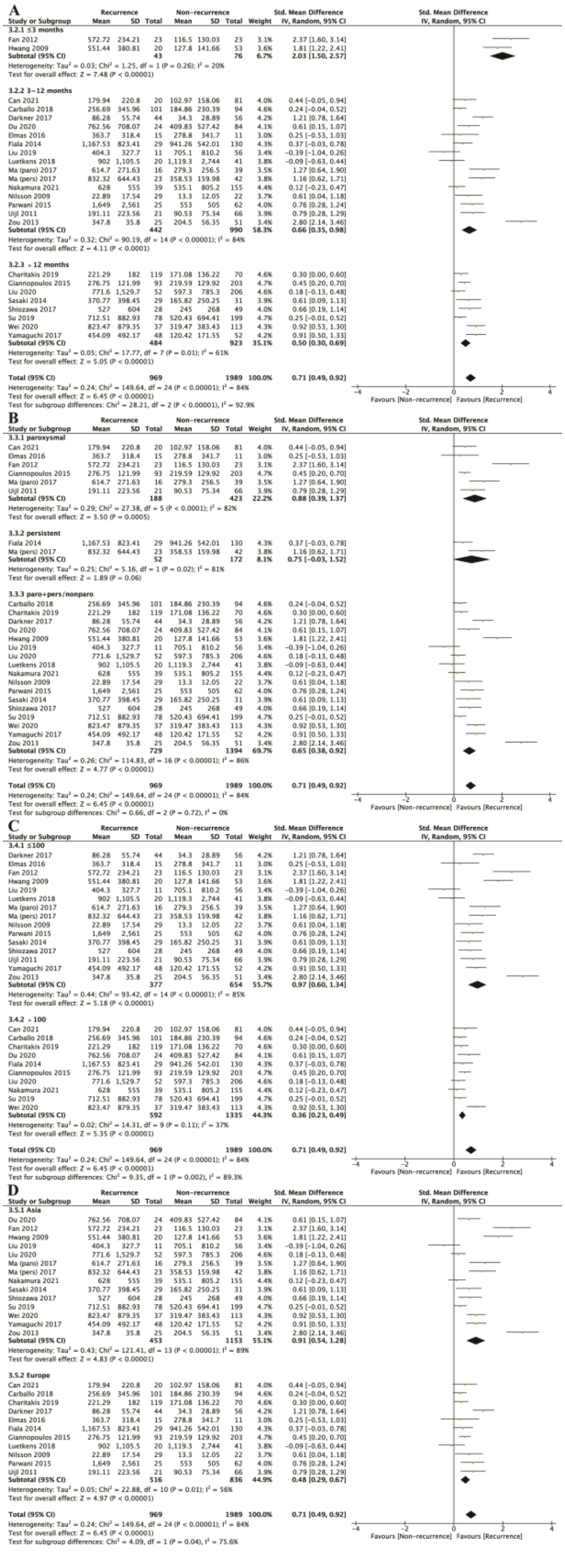

Supplement: Supplementary file 6 [file medi-102-e33704-s006.pdf]
