# Supplementary material for: Natriuretic peptides as predictors for atrial fibrillation recurrence after catheter ablation: A meta-analysis
Source: Medicine (Baltimore). 2023 May 12;102(19):e33704. doi: 10.1097/MD.0000000000033704 (PMC10174372; doi:10.1097/MD.0000000000033704)

Figure S6 Sensitive analysis of the association of NT-proBNP with the post-ablation AF recurrence.

	Recurrence			Non-recurrence				Std. Mean Difference
Study or Subgroup	Mean	SD	Total	Mean	SD	Total	Weight	IV, Random, 95% CI
Can 2021	179.94	220.8	20	102.97	158.06	81	4.6%	0.44 [−0.05, 0.94]
Carballo 2018	256.69	345.96	101	184.86	230.39	94	5.5%	0.24 [−0.04, 0.52]
Charitakis 2019	221.29	182	119	171.08	136.22	70	5.4%	0.30 [0.00, 0.60]
Darkner 2017	86.28	55.74	44	34.3	28.89	56	4.9%	1.21 [0.78, 1.64]
Du 2020	762.56	708.07	24	409.83	527.42	84	4.7%	0.61 [0.15, 1.07]
Elmas 2016	363.7	318.4	15	278.8	341.7	11	0.0%	0.25 [−0.53, 1.03]
Fan 2012	572.72	234.21	23	116.5	130.03	23	0.0%	2.37 [1.60, 3.14]
Fiala 2014	1,167.53	823.41	29	941.26	542.01	130	5.0%	0.37 [−0.03, 0.78]
Giannopoulos 2015	276.75	121.99	93	219.59	129.92	203	5.6%	0.45 [0.20, 0.70]
Hwang 2009	551.44	380.81	20	127.8	141.66	53	4.1%	1.81 [1.22, 2.41]
Liu 2019	404.3	327.7	11	705.1	810.2	56	0.0%	−0.39 [−1.04, 0.26]
Liu 2020	771.6	1,529.7	52	597.3	785.3	206	5.4%	0.18 [−0.13, 0.48]
Luetkens 2018	902	1,105.5	20	1,119.3	2,744	41	4.4%	−0.09 [−0.63, 0.44]
Ma (paro) 2017	614.7	271.63	16	279.3	256.5	39	4.0%	1.27 [0.64, 1.90]
Ma (pers) 2017	832.32	644.43	23	358.53	159.98	42	4.3%	1.16 [0.62, 1.71]
Nakamura 2021	628	555	39	535.1	805.2	155	5.2%	0.12 [−0.23, 0.47]
Nilsson 2009	22.89	17.54	29	13.3	12.05	22	4.2%	0.61 [0.04, 1.18]
Parwani 2015	1,649	2,561	25	553	505	62	4.6%	0.76 [0.28, 1.24]
Sasaki 2014	370.77	398.45	29	165.82	250.25	31	4.5%	0.61 [0.09, 1.13]
Shiozawa 2017	527	604	28	245	268	49	4.7%	0.66 [0.19, 1.14]
Su 2019	712.51	882.93	78	520.43	694.41	199	5.5%	0.25 [−0.01, 0.52]
Uijl 2011	191.11	223.56	21	90.53	75.34	66	4.5%	0.79 [0.28, 1.29]
Wei 2020	823.47	879.35	37	319.47	383.43	113	5.1%	0.92 [0.53, 1.30]
Yamaguchi 2017	454.09	492.17	48	120.42	171.55	52	0.0%	0.91 [0.50, 1.33]
Zou 2013	347.8	35.8	25	204.5	56.35	51	3.8%	2.80 [2.14, 3.46]
**Total (95% CI)**			**872**			**1847**	**100.0%**	**0.69 [0.48, 0.91]**

Heterogeneity: $Tau^2 = 0.20$; $Chi^2 = 116.36$, df = 20 ($P < 0.00001$); $I^2 = 83\%$
Test for overall effect: Z = 6.30 ($P < 0.00001$)

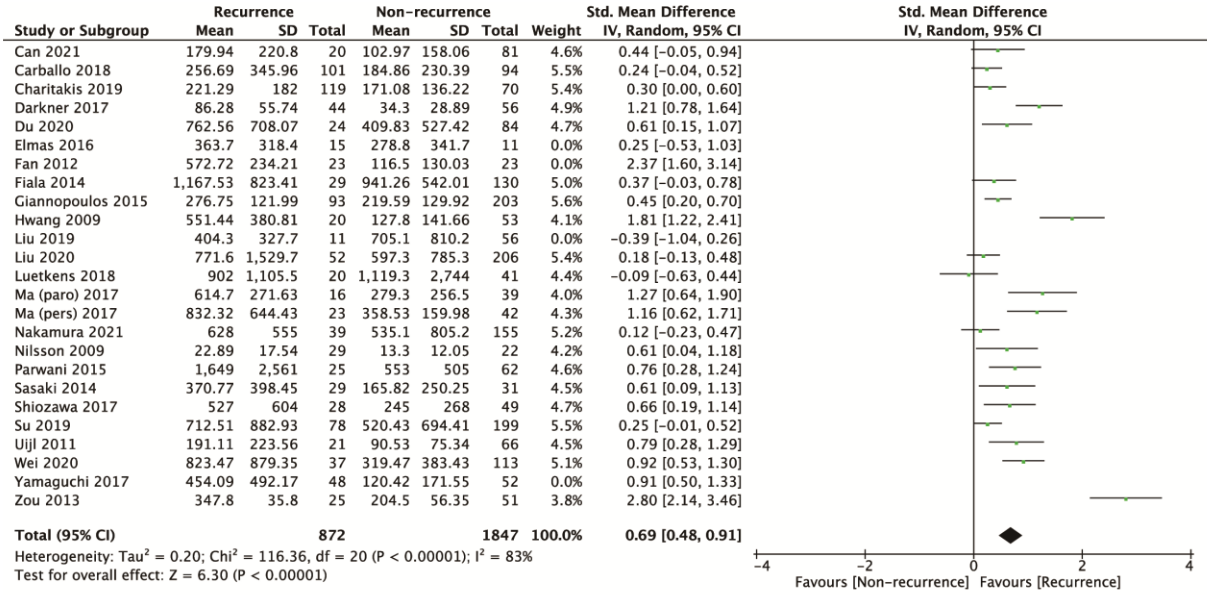

Supplement: Supplementary file 7 [file medi-102-e33704-s007.pdf]
