# Supplementary material for: Natriuretic peptides as predictors for atrial fibrillation recurrence after catheter ablation: A meta-analysis
Source: Medicine (Baltimore). 2023 May 12;102(19):e33704. doi: 10.1097/MD.0000000000033704 (PMC10174372; doi:10.1097/MD.0000000000033704)

Figure S7 Sensitive analysis of the association of MR-proANP with the post-ablation AF recurrence.

Study or Subgroup	Recurrence Mean	Recurrence SD	Recurrence Total	Non-recurrence Mean	Non-recurrence SD	Non-recurrence Total	Weight	Std. Mean Difference IV, Random, 95% CI
Badoz 2021	204.79	83.59	34	103.24	67.95	71	32.6%	1.37 [0.92, 1.83]
Charitakis 2019	139.64	77.42	119	118.3	53.91	70	34.7%	0.31 [0.01, 0.60]
Darkner 2017	205.08	107.69	44	95.28	43.33	56	32.7%	1.39 [0.95, 1.83]
Elmas 2016	143.1	67.8	15	108.4	49	11	0.0%	0.55 [−0.24, 1.35]
**Total (95% CI)**			**197**			**197**	**100.0%**	**1.01 [0.23, 1.79]**

Heterogeneity: $Tau^2 = 0.44$; $Chi^2 = 23.80$, df = 2 ($P < 0.00001$); $I^2 = 92\%$
Test for overall effect: $Z = 2.53$ ($P = 0.01$)

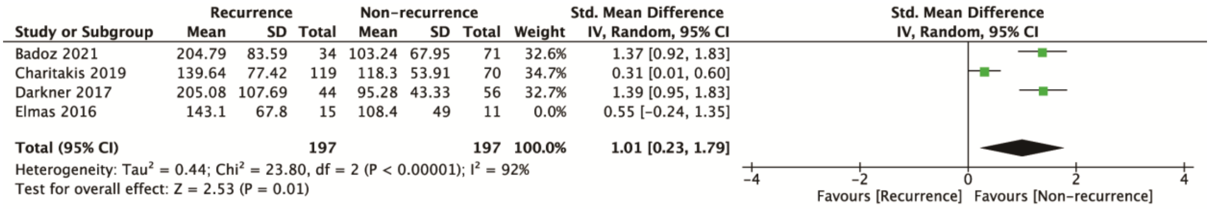

Supplement: Supplementary file 8 [file medi-102-e33704-s008.pdf]
