# Supplementary material for: Natriuretic peptides as predictors for atrial fibrillation recurrence after catheter ablation: A meta-analysis
Source: Medicine (Baltimore). 2023 May 12;102(19):e33704. doi: 10.1097/MD.0000000000033704 (PMC10174372; doi:10.1097/MD.0000000000033704)
Supplement: Supplementary file 10 [file medi-102-e33704-s010.pdf]

Figure S9 Trim and fill analysis of NT-proBNP.

```
Meta-analysis

       |  Pooled      95% CI         Asymptotic      No. of
Method |     Est   Lower   Upper  z_value  p_value   studies
-------+----------------------------------------------------
Fixed  |   0.539   0.457   0.622   12.784    0.000     25
Random |   0.707   0.485   0.928    6.252    0.000

Test for heterogeneity: Q= 160.109 on 24 degrees of freedom (p= 0.000)
Moment-based estimate of between studies variance =  0.258

Trimming estimator: Linear
Meta-analysis type: Random-effects model

iteration |  estimate    Tn    # to trim     diff
----------+--------------------------------------
    1     |     0.707    143          0        325
    2     |     0.707    143          0          0

Note: no trimming performed; data unchanged

Filled
Meta-analysis

       |  Pooled      95% CI         Asymptotic      No. of
Method |     Est   Lower   Upper  z_value  p_value   studies
-------+----------------------------------------------------
Fixed  |   0.539   0.457   0.622   12.784    0.000     25
Random |   0.707   0.485   0.928    6.252    0.000

Test for heterogeneity: Q= 160.109 on 24 degrees of freedom (p= 0.000)
Moment-based estimate of between studies variance =  0.258
```
